# Long non-coding RNA NNT-AS1 positively regulates NPM1 expression to affect the proliferation of estrogen-mediated endometrial carcinoma by interacting

**DOI:** 10.7150/jca.62630

**Published:** 2022-01-01

**Authors:** Jie Shen, Zhilin Yuan, Jingjing Sheng, Xiaoping Feng, Hao Wang, Yanli Wang, Yunxiao Zhou

**Affiliations:** 1 Department of Gynecology, The First Affiliated Hospital, College of Medicine, Zhejiang University, Hangzhou, Zhejiang, China.; 2 Department of Obstetrics and Gynecology, the Fourth Affiliated Hospital, College of Medicine, Zhejiang University, Hangzhou, Zhejiang, China.; 3 Department of Gynecology, Yiwu Central Hospital, Yiwu, Zhejiang, China.; 4 Department of Pathology, The First Affiliated Hospital, College of Medicine, Zhejiang University, Hangzhou, Zhejiang, China.

**Keywords:** NNT-AS1, miR-30c, NPM1, endometrial carcinoma, estrogen

## Abstract

**Objective:** This study aims to investigate the mechanism of long non-coding RNA NNT-AS1 in the proliferation of estrogen-mediated endometrial carcinoma (EC).

**Materials and methods:** NNT-AS1, miR-30c, and Nucleophosmin 1 (NPM1) expressions were measured by quantitative real-time PCR and Western blotting. Cell Counting Kit-8 assay and 5-Ethynyl-2'-deoxyuridine (EdU) assay were used to detect the viability and proliferation of Ishikawa and HEC-1-A cells, respectively. RNA immunoprecipitation assay was used to confirm the interaction between NNT-AS1 and miR-30c. Luciferase reporter assay was performed to confirm the interaction between miR-30c and NPM1.

**Results:** NNT-AS1 and NPM1 expressions in EC tissues and cell lines were higher than in benign endometrium and normal endometrial epithelial cells (EECs). miR-30c expression in EC tissues and cell lines was lower than in benign endometrium and normal EECs. NNT-AS1 interacted with miR-30c, and miR-30c negatively regulated NPM1 expression. Overexpression of NNT-AS1 increased NPM1 expression in EC cells, while overexpression of miR-30c reversed the effect. NNT-AS1 interference inhibited the mRNA level of NPM1, while the miR-30c inhibitor reversed the result. Estradiol (E_2_) promoted the proliferation of EC cells, small interfering RNA (siRNA) against NNT-AS1 inhibited EC cell proliferation, miR-30c inhibitor promoted cell proliferation, and NPM1 siRNA inhibited cell proliferation. E_2_ increased tumor volume, and NNT-AS1 interference reduced tumor volume *in vivo*.

**Conclusion:** NNT-AS1 promoted the proliferation of estrogen-mediated EC by regulating miR-30c/NPM1.

## Introduction

Endometrial carcinoma (EC) is one of the three most common female genital tract malignancies. Every year, health authorities record 287,100 new cases of EC worldwide, and these continue to increase in recent years 1. Based on differences in clinical symptoms and epidemiology, there are two subtypes of EC. Type I endometrioid cancer is related to estrogen, which commonly occurs in perimenopausal women. On the other hand, Type II non-endometrioid cancer is unrelated to estrogen. It occurs in the atrophic endometrium of older women 2, which shows that estrogen is essential for developing Type I EC. Type I EC accounts for 80% of all ECs 2. In China, the onset age is getting younger and younger, especially in developed areas 3. Therefore, it is critical to explore the underlying mechanisms involved in EC development, primarily Type I EC, which could be helpful in making an early diagnosis and decreasing mortality.

Nucleophosmin 1 (NPM1) is a nucleolar phosphoprotein and a member of Nucleophosmin, proven to be involved in centrosome duplication, cell apoptosis, cell differentiation, cell cycle progression, and maintaining genomic stability. All of these suggest that NPM1 participates in tumorigenesis 4. As reported, a high level of estrogen and estrogen receptors is responsible for the progression of Type I EC 5. Our previous study found that the expression of NPM1 gradually increased as the clinical stages of EC progressed, and that estrogen increased NPM1 levels via estrogen receptor-α (ERα) signaling 6. Therefore, NPM1 plays an oncogenic role in estrogen-mediated EC. However, the molecular mechanisms underlying the elevation of NPM1 in estrogen-mediated EC remain unclear.

MicroRNAs (miRNAs) are small non-coding RNAs that usually contain 21-24 nucleotides and regulate the expression of target mRNAs at the post-transcriptional level, which play essential roles in tumorigenesis and the progression of EC 7, 8. Several reports have found that EC cells and EC tissue abnormally expressed miR-30c by targeting metastasis-associated gene-1 8-10. Moreover, researchers have found that in acute myeloid leukemia, miR-30c was NPM1 mutation-associated microRNA that might contribute to chemosensitivity 11. Thus, we assumed that there might be a relationship between miR-30c and NPM1 in EC.

Long non-coding RNAs (lncRNAs, > 200 nt in length) have functional importance and can regulate the activation and inactivation of genes 12. Studies have demonstrated that lncRNA could be a useful biomarker in cancers. For example, esophageal squamous cell carcinoma (ESCC) upregulated lncRNA ATB, which promoted the malignancy of ESCC via miR-200b/Kindlin-2 13. Colon cancer cells overexpressed LncRNA H19, which induced resistance to 1,25(OH)_2_D3 by targeting the vitamin D receptor (VDR) 14. LncRNA NNT-AS1 is a newly found lncRNA, and its role in cancers is still unknown. In 2017, Wang *et al.* firstly reported that NNT-AS1 promoted proliferation and migration of colorectal cancer cells 15. Hua *et al.* found that NNT-AS1 was overexpressed in cervical cancer and promoted cell proliferation and invasion via the Wnt/β-catenin pathway 16. Thus, we assumed that EC might abnormally express NNT-AS1. Also, the bioinformatics software (http://carolina.imis.athena-innovation.gr/diana_tools) predicted that there were binding sites between NNT-AS1 and miR-30c. Herein, we speculated that NNT-AS1 regulated NPM1 expression to affect the progress of estrogen-mediated EC via miR-30c.

In this study, we aimed to determine whether NNT-AS1 played a role in the development of EC and explored its possible mechanism in regulating the process of EC. We detected the expressions of NNT-AS1, miR-30c, and NPM1 in EC tissues and EC cells and the potential role of NNT-AS1 in regulating the viability of EC cells. Eventually, we found that NNT-AS1 participated in the process of EC via miR-30c/NPM1.

## Materials and methods

### Samples and cell culture

Forty EC tissues and 40 benign endometrium tissues were obtained from patients who underwent operations at the First Affiliated Hospital, College of Medicine, Zhejiang University, from 2016 to 2021. The major inclusion criteria include: patients with histologically confirmed EC of all histological subtypes. Benign endometrium tissues were collected from histologically confirmed normal para-cancer tissues. The exclusion criteria include: patients with uterine sarcomas (except for carcinosarcomas) or visual extrauterine disease. The Ethics Committee approved this project of The First Affiliated Hospital, College of Medicine, Zhejiang University. All patients signed informed consent forms before the study. The histological diagnosis and FIGO staging of EC were performed by two experienced pathologists. No patients received preoperative chemotherapy or radiation therapy.

Endometrial epithelial cells (EECs) and human EC cell lines Ishikawa and HEC-1-A cells were purchased from the American Type Culture Collection (ATCC, Manassas, VA, USA). We then cultured these in DMEM/F12 (Gibco, Waltham, MA, USA) supplemented with 10% fetal bovine serum (Gibco, Waltham, MA, USA), penicillin (100 U/ml), and streptomycin (100 mm/ml) in a 5% CO_2_ incubator under 37 °C until cells became 80-90% confluent from passages 4-6. To determine estradiol (E2) regulation on cell viability, we treated cells with 17β-estradiol (E_2_, 1 nM, Sigma-Aldrich, St Louis, MO, USA) for 24 h in 6-well plates.

### Cell transfection

Using Invitrogen (Waltham, MA, USA), we synthesized miR-30c mimic, miR-30c inhibitor, NNT-AS1-overexpressing vector (pcDNA-NNT-AS1), NNT-AS1 small interfering RNA (siRNA-NNT-AS1), and their negative controls (NC). We then transfected them into cells using Lipofectamine 2000 (Invitrogen, Waltham, MA, USA) for 48 h. We served the non-transfected Ishikawa and HEC-1-A cells in the culture medium as negative controls.

### Quantitative real-time PCR (qRT-PCR)

Using TRIzol Reagent (Invitrogen, Waltham, MA, USA), we isolated the total RNA from EC tissues, benign endometrium tissues, EECs, Ishikawa cells, and HEC-1-A cells according to the manufacturer's instructions. The PowerUp™ SYBR™ Green Master Mix (Applied Biosystems, USA) detected the expressions of NNT-AS1 and miR-30c, and QuantStudio® 3 RCR Real-Time PCR systems (Applied Biosystems, Foster City, CA, USA) performed qRT-PCR. Using the comparative method 2^-ΔΔCt^, we determined the relative NNT-AS1 and miR-30c expressions and amplified U6 and GAPDH as an internal control. The primers used were shown as follows: NNT-AS1 forward, 5'-ACGTG CAGACAACATCTACCT-3', reverse, 5'-TACAACACCTTCCCGCAT-3'; miR-30c forward, 5'-TGTGTTTTTATTGTTTTTGTTGTCCCA-3', reverse, 5'-GGGACAG AACAGGTTAATGGGAA-3'; NPM1 mRNA forward, 5'-GGAGGTGGTAGCAAG GTTCC-3', reverse, 5'-TTCACTGGCGCTTTTTCTTCA-3'; U6 forward 5'-CTCGCTTCGGCAGCACA-3', reverse 5'-ACGCTTCACGAATTTGCGT-3'; GAPDH forward 5'-AATGGGCAGCCGTTAGGAAA-3', reverse 5'-GCGCCCAAT ACGACCAAATC-3'.

### Western blotting

Using RIPA buffer (Beyotime Biotechnology, China), we extracted the proteins from EC tissues, benign endometrium tissues, EECs, Ishikawa cells, and HEC-1-A cell lines. Meanwhile, the BCA Protein Assay kit (Pierce Biotechnology, USA) detected protein concentrations. We isolated equal amounts of protein samples (50 μg) in 10% SDS-polyacrylamide gel electrophoresis (SDS-PAGE) and then transferred these to polyvinylidene difluoride (PVDF) membranes (Invitrogen). We blocked the membrane in 5% non-fat dried milk, then probed it with primary antibody anti-NPM1 (1:1000, ab10530, Abcam, Cambridge, UK), anti-β-actin (1:1000, #3700S, Cell Signaling Technology, USA), and the secondary horseradish peroxidase-conjugated antibody (1:2000, ab6728, Abcam, Cambridge, UK). An ECL Western Blotting System (GE Healthcare, USA) detected proteins, and we used β-actin as a control protein to quantify the expression of related proteins.

### Cell viability assay

Using a cell counting kit-8 (CCK-8) (EnoGene, China), we detected the cell viability of Ishikawa and HEC-1-A cells according to the manufacturer's instructions. A microplate reader (QIAGEN) read the optical density (OD) value for each well at 450 nm.

### Cell proliferation assay

The Cell-Light 5-Ethynyl-2'-deoxyuridine (EdU) Apollo488 *In vitro* Kit (RiboBio Technology, Guangzhou, Guangdong, China) detected cell proliferation. We inoculated the cells in the logarithmic growth phase 4×10^3^~1×10^5^ cells per well in 96-well plates and cultured them to the expected growth stage. We diluted the EdU solution with cell complete medium at a ratio of 1000:1 to prepare an appropriate amount of 50 μM EdU medium. Then, we added 100 μL of 50 μM EdU medium to each well and incubated them for 2 h before discarding the medium. We washed the cells with PBS twice (5 min each time). We added a cell fixation solution (50 μL) to each well and incubated cells for 30 min at room temperature after discarding the fixation solution. Then, we added 100 μL of PBS to each well and washed the cells in a decolorizing shaker for 5 min. After that, we added 100 μL of penetrant (0.5% TritonX-100 in PBS) to each well and incubated cells on a decolorizing shaker for 10 min before washing them with PBS once for 5 min. We added the Apollo 488 staining reaction solution (100 μL) to each well. After incubating for 30 minutes on a decolorizing shaker in the dark at room temperature, we discarded the staining reaction solution. Then, we added 100 μL of penetrant (0.5% TritonX-100 in PBS). After disposing of the penetrant solution, we diluted the reagent F (Hoechst 33342) using deionized water at a ratio of 100:1 and stored it in the dark. Then, we added 100 μL Hoechst 33342 to each well. After incubating the reaction solution in the dark for 30 min, we discarded the staining reaction solution and then washed the cells with 100 μL of PBS three times. We observed the cells under a fluorescence microscope (Olympus, Tokyo, Japan) at an excitation wavelength of 490 nm for Apollo 488 and 350 nm for Hoechst 33342.

### Cell apoptosis assay

We detected cell apoptosis using the Annexin V-FITC/PI Apoptosis Detection Kit (Elabscience, Wuhan, Hubei, China). We centrifuged the cells at 300 g for 5 min and discarded the supernatant. We collected the cells and washed them once with PBS. Then, we gently resuspended and counted the cells. We centrifuged the resuspended cells (1×10^5^) at 300 g for 5 min before the wash with PBS. We added the diluted Annexin V Binding Buffer working solution (500 μL) to the cells. After the cell resuspension, we added 5 μL of Annexin V FITC and 5 μL of PI staining solution to the cell suspension. We incubated the cells in the dark for 15-20 min at room temperature. After the reaction, we immediately tested the cells under a flow cytometer (FACSCanto II; BD Biosciences, San Jose, CA, USA).

### Dual-luciferase reporter assays

RiboBio Technology (Guangzhou, Guangdong, China) established the pMIR plasmid (400 ng) containing wild type 3' untranslated region (3'UTR) of NPM1 mRNA (NPM1-WT) or mutant 3'UTR of NPM1 mRNA (NPM1-Mut). We transfected them into Ishikawa and HEC-1-A cells with 40 ng pRL-TK plasmid (Promega, Madison, WI, USA). We transfected miR-30c mimic, miR-30c inhibitor, or NC into Ishikawa and HEC-1-A cells for 48 h. Finally, we collected Ishikawa and HEC-1-A cells to measure luciferase activity using a dual Glo^TM^ Luciferase Assay System (Promega, Madison, WI, USA).

### RNA immunoprecipitation (RIP) assays

The bioinformatics software (http://carolina.imis.athena-innovation.gr/diana_tools) predicted that there were binding sites between NNT-AS1 and miR-30c. According to the manufacturer's instructions, we used a Magna RIPTM RNA-Binding Protein Immunoprecipitation Kit (Millipore, Temecula, CA, USA) for RIP experiments. We purchased the antibody for RIP assays of endogenous Argonaute 2 (AGO2) from Cell Signaling Technology (Danvers, MA, USA). IP-western assessed AGO2, and qRT-PCR detected NNT-AS1 and miR-30c.

### RNA pull-down assays

We used Biotin-labeled NNT-AS1 as a probe to determine AGO2 by Western blotting and miR-30c by qRT-PCR. We transcribed the Biotin-labeled NNT-AS1 with the Biotin RNA Labeling Mix (Roche, Basel, Switzerland) and T7 RNA polymerase, and purified it with a high Pure FFPET RNA Isolation Kit (Roche, Basel, Switzerland). Then, we mixed the cell nuclear protein (1 mg) extract with biotinylated RNA biotin-labeled RNAs (100 pmol) and added streptavidin agarose beads (Invitrogen, Waltham, MA, USA) before incubating for 1 h at room temperature. We washed the beads three times and boiled them in SDS buffer. We used standard western blotting to detect proteins binding to the retrieved protein.

### Xenograft experiments

We purchased 18 female BALB/c nude mice from Shanghai Lab Animal Research Center (Shanghai, China). We kept them in pathogen-free conditions and divided them into three groups: sh-control (n = 6), E2 + sh-control (n = 6), and E2 + shRNA-NNT-AS1 (n = 6). We transfected Ishikawa cells with lentivirus containing small hairpin RNA against NNT-AS1 (shRNA-NNT-AS1) or control shRNA (sh-control), established by Hanbio Technology (Shanghai, China). We then injected the stable transfection Ishikawa cells (5×10^6^) into the subscapular region of the nude mice. Four weeks later, we took the subcutaneous tumors out for orthotopic implantation, and we removed the ovaries of all mice. We immediately implanted tumor samples (about 1 mm^3^) and fixed them onto the posterior face of the uterus. Ninety-day release pellets 17β-estradiol (0.72 mg/pellet) supplied estrogen to the mice subcutaneously. Six weeks later, we sacrificed the animals, and we took out the tumors. The Ethics Committee approved all procedures.

### Statistical analysis

We used SPSS 18.0 software for data analysis. Mean ± SD expressed the result. We performed all experiments in triplicate. The student's t-test analyzed the differences between the two groups. One-way analysis of variance (ANOVA) followed by the Tukey's post hoc test examined the differences among multiple groups, with P < 0.05 considered statistically significant.

## Results

### Expression of NNT-AS1, miR-30c, and NPM1 in EC tissues and cell lines

**Figure 1:**
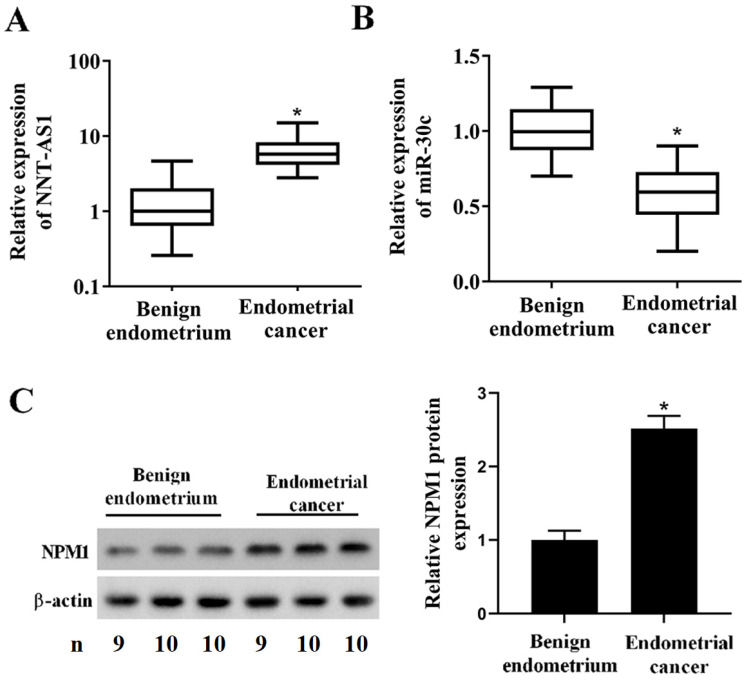
** Expression of NNT-AS1, miR-30c, and NPM1 in EC tissues and benign endometrium tissues.** Forty EC tissues and paired benign endometrium tissues were obtained from patients who underwent operations. **A.** NNT-AS1 expression in 40 paired tissues was detected using qRT-PCR. GAPDH was used as the internal control. **B.** miR-30c expression in 40 paired tissues was detected using qRT-PCR. U6 was used as the internal control. Each experiment has three technological duplications. **C.** The protein level of NPM1 in mixture samples of 29 paired tissues (n = 9, 10, and 10) was detected using western blotting. β-actin was used as the internal control. Student's t-test was used for statistical analysis between the benign endometrium and the EC groups. *p < 0.05, compared with benign endometrium.

**Figure 2:**
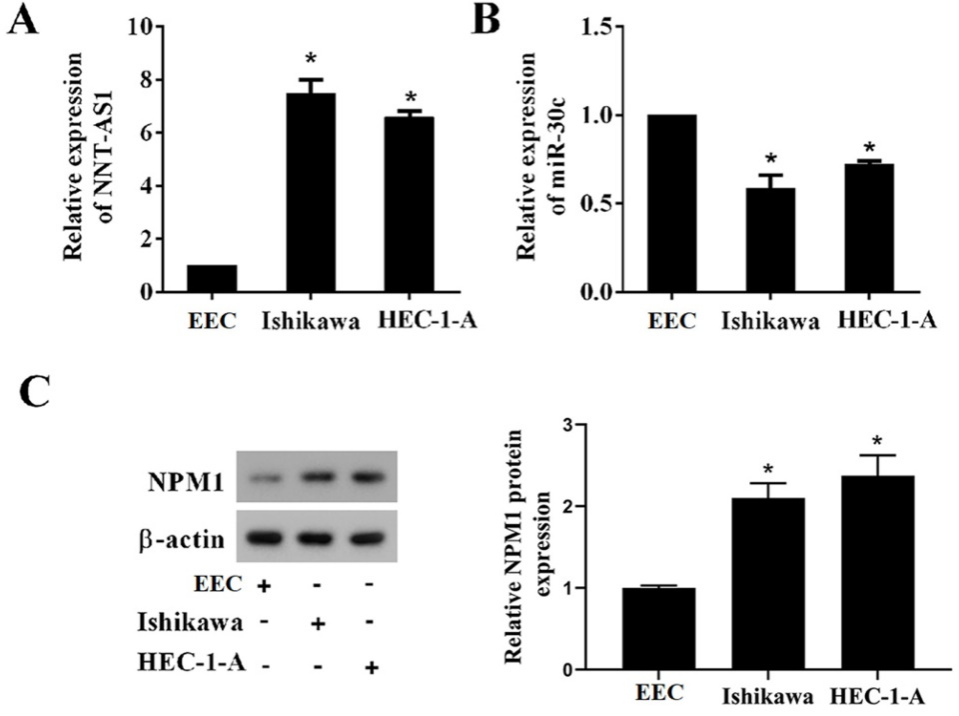
** Expression of NNT-AS1, miR-30c, and NPM1 in EC cells and endometrial epithelial cells (EECs).** A. NNT-AS1 expression was detected using qRT-PCR. GAPDH was used as the internal control. B. miR-30c expression was detected using qRT-PCR. U6 was used as the internal control. C. The protein level of NPM1 was detected using western blotting. β-actin was used as the internal control. Each experiment has three biological duplications. One-way ANOVA followed by Tukey's post hoc test was used for statistical analysis. *p < 0.05, compared with EEC.

To determine whether EC tissues and cells abnormally expressed NNT-AS1, miR-30c, and NPM1, we used qRT-PCR and Western blotting to measure their expressions. As shown in **Figure 1A**, NNT-AS1 expression in EC tissues was higher than in benign endometrium tissues. miR-30c expression in EC tissues was lower than in benign endometrium tissues (**Figure 1B**). Meanwhile, the higher expression of NNT-AS1 in endometrium tissues was related to the higher FIGO stage and pathology classification (**Table 1**). The protein level of NPM1 in EC tissues was higher than in benign endometrium tissues (**Figure 1C**). Next, we accessed the levels of NNT-AS1, miR-30c, and NPM1 in EC cell lines (Ishikawa and HEC-1-A cells) and normal EECs, and found that NNT-AS1 expression in EC cells was higher than in EECs (**Figure 2A**). miR-30c expression in EC cells was lower than in EECs (**Figure 2B**). The protein level of NPM1 in EC cells was higher than in EECs (**Figure 2C**).

### Interference with NNT-AS1 inhibited the proliferation of estrogen-induced EC cells

**Figure 3:**
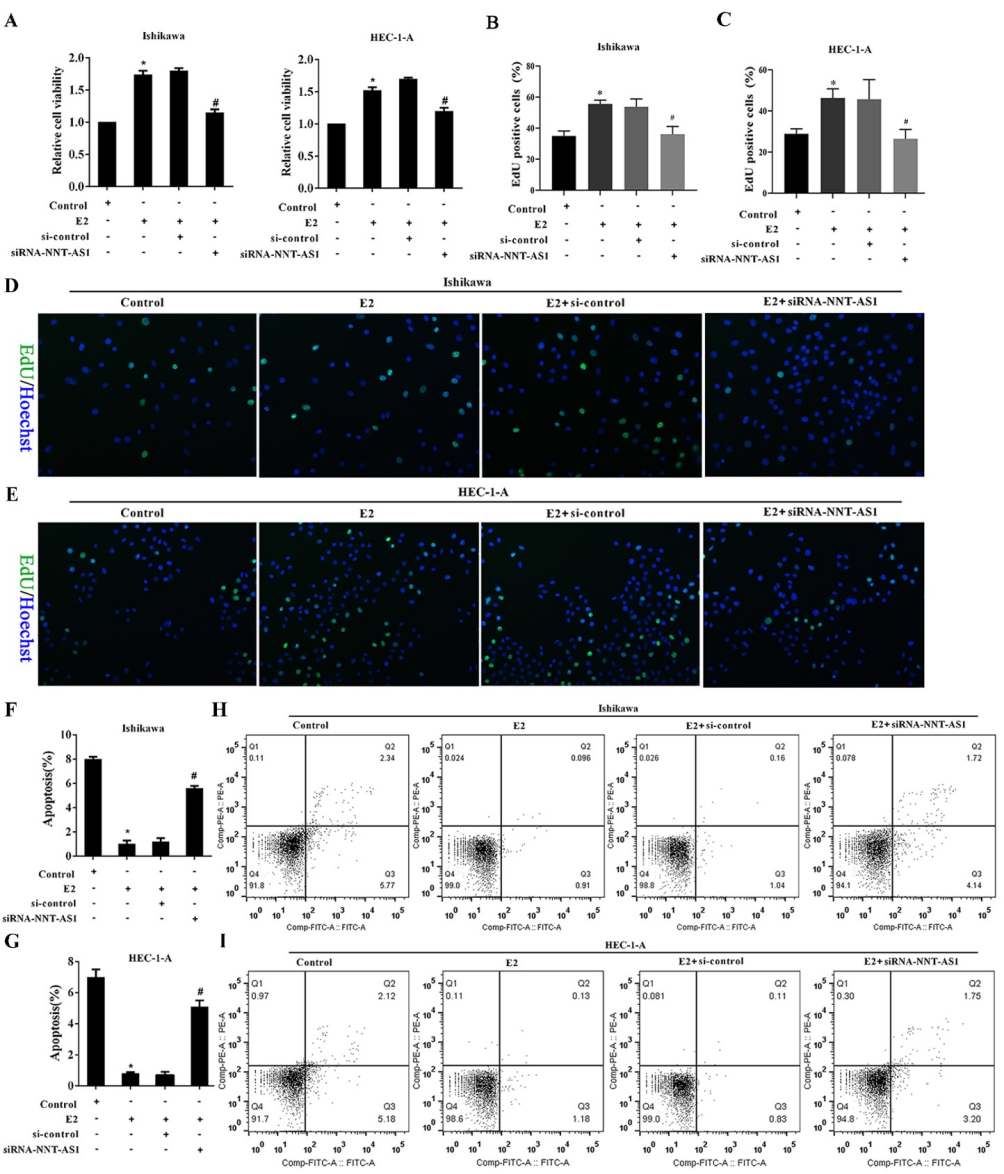
** Interference with NNT-AS1 inhibited the proliferation of estrogen-induced EC cells.** Ishikawa and HEC-1-A cells were transfected with small interfering RNAs (si-control and siRNA-NNT-AS1) for 48 h before E_2_ treatment. **A.** The cell viability was detected by Cell Counting Kit-8 assay. **B.** The cell proliferation was detected using the EdU assay. **C-F.** The cell apoptosis was observed by Flow cytometry. Each experiment has three biological duplications. One-way ANOVA followed by Tukey's post hoc test was used for statistical analysis. *p < 0.05, compared with Control. ^#^p < 0.05, compared with E2 + si-control.

To investigate the effect of NNT-AS1 on the proliferation of E_2_-induced EC cells, we observed cell proliferation after transfecting siRNA-NNT-AS1 into E_2_-induced EC cells. E_2_ promoted the cell viability and proliferation of Ishikawa and HEC-1-A cells, while siRNA-NNT-AS1 reversed these effects (**Figure 3A**-**B**). We also observed cell apoptosis after transfecting siRNA-NNT-AS1 into E_2_-induced EC cells, and we found that E_2_ decreased the apoptotic rate of Ishikawa and HEC-1-A cells, while siRNA-NNT-AS1 reversed this effect (**Figure 3C**-**F**).

### NNT-AS1 interacted with miR-30c

**Figure 4:**
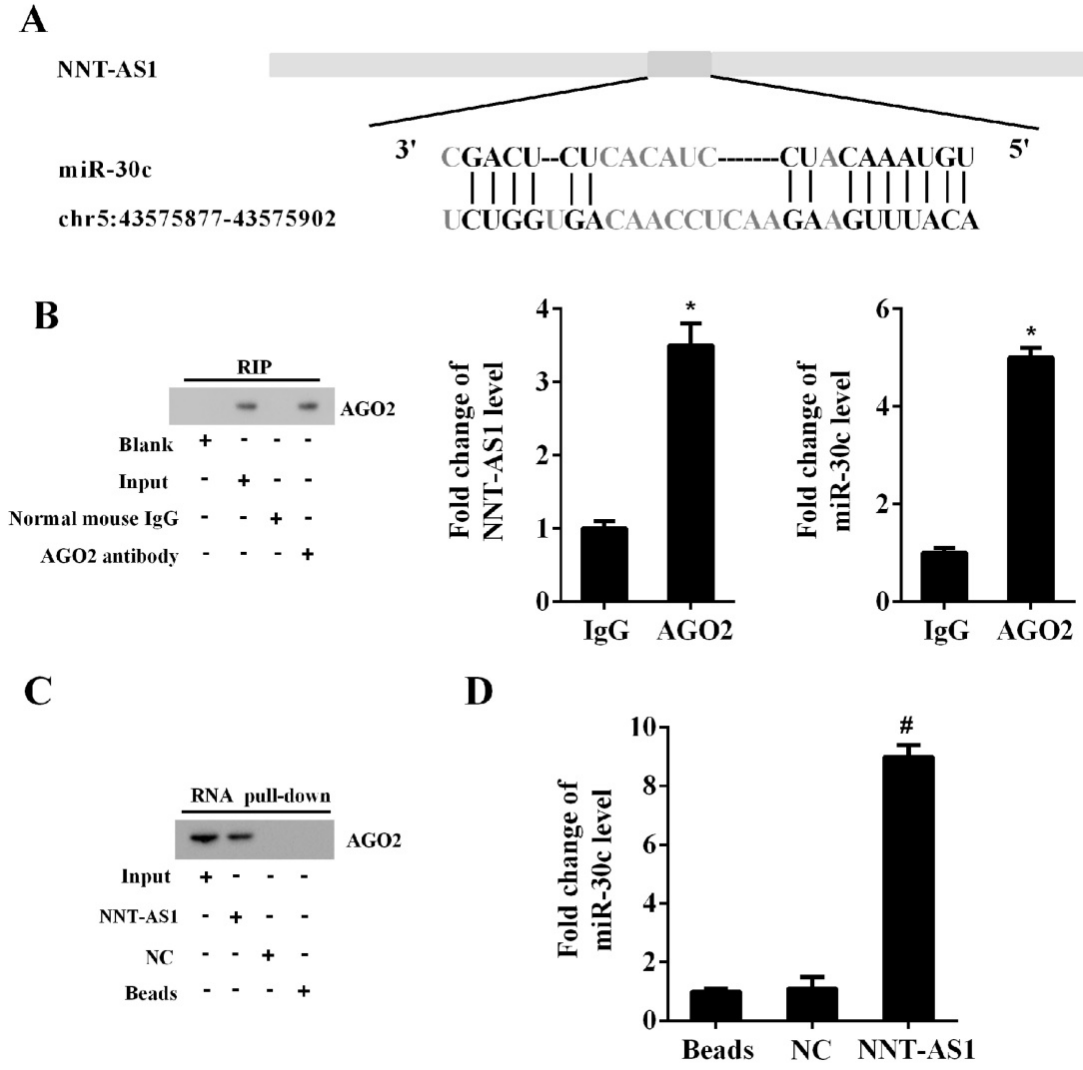
** The interaction between NNT-AS1 and miR-30c. A.** Bioinformatics software predicted the binding sites between NNT-AS1 and miR-30c. **B.** NNT-AS1 and miR-30c were accumulated in AGO2 immunoprecipitants. AGO2 was detected by Western blotting, and RNA levels of NNT-AS1 and miR-30c were detected by qRT-PCR. **C.** The expression of AGO2 in the NNT-AS1 pulled-down complex was detected using Western blotting. **D.** The expression of miR-30c in the NNT-AS1 pulled-down complex was detected using qRT-PCR. Each experiment has three biological duplications. Student's t-test was used for statistical analysis between 2 groups. One-way ANOVA followed by Tukey's post hoc test was used for statistical analysis among multiple groups. *p < 0.05, compared with IgG. ^#^p < 0.05, compared with NC.

The Bioinformatics software (http://carolina.imis.athena-innovation.gr/diana_tools) predicted the potential interactions between NNT-AS1 and miR-30c (**Figure 4A**). RIP assay and RNA pull-down assay confirmed the interaction between NNT-AS1 and miR-30c. As shown in **Figure 4B**, the RIP assay confirmed the enrichment of NNT-AS1 and miR-30c in AGO2 immunoprecipitants. Meanwhile, the RNA pull-down assay confirmed the enrichment of AGO2 protein and miR-30c in the NNT-AS1 pulled-down complex (**Figure 4C-D**).

### miR-30c negatively regulated NPM1 expression

**Figure 5:**
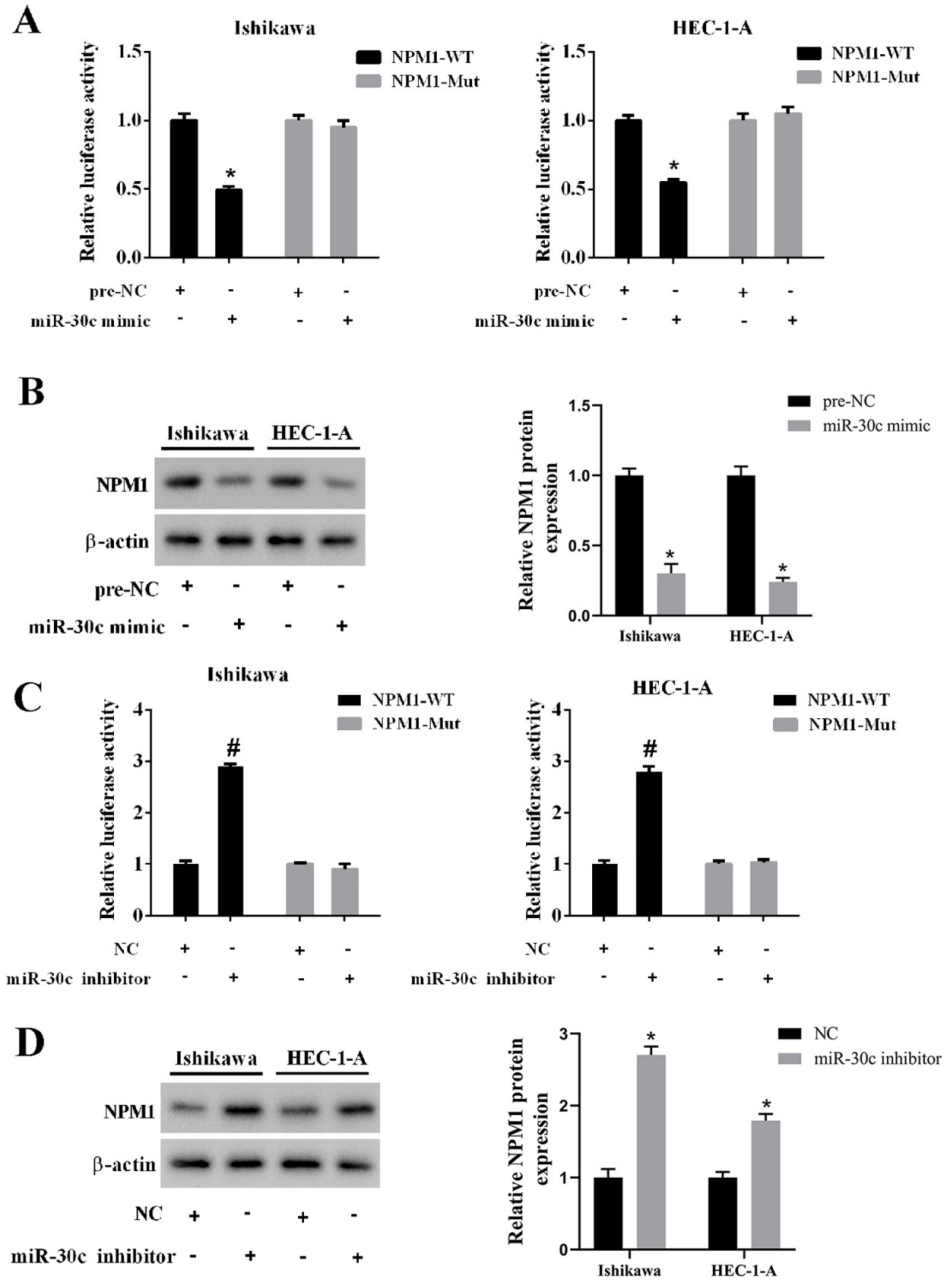
** Regulation of miR-30c on NPM1 expression. A.** Ishikawa and HEC-1-A cells were transfected with miR-30c mimic or the negative control (pre-NC) with luciferase reporter gene vector (NPM1-WT or NPM1-Mut). The luciferase activity was detected using the dual luciferase reporter assay. **B.** Ishikawa and HEC-1-A cells were transfected with miR-30c mimic or the negative control (pre-NC). The protein level of NPM1 was detected using Western blotting. β-actin was used as the internal control. **C.** Ishikawa and HEC-1-A cells were transfected with miR-30c inhibitor or the negative control (NC) with luciferase reporter gene vector (NPM1-WT or NPM1-Mut). The luciferase activity was detected using the dual luciferase reporter assay. **D.** Ishikawa and HEC-1-A cells were transfected with miR-30c inhibitor or the negative control (NC). The protein level of NPM1 was detected using Western blotting. β-actin was used as the internal control. Each experiment has three biological duplications. One-way ANOVA followed by Tukey's post hoc test was used for statistical analysis. *p < 0.05, compared with pre-NC. ^#^p < 0.05, compared with NC.

To confirm the relationship between miR-30c and NPM1, we conducted a luciferase reporter gene assay. We found that the overexpression of miR-30c inhibited luciferase activity in the wild type 3'UTR group, while it did not affect luciferase activity in the mutant 3'UTR group (**Figure 5A**). In addition, the overexpression of miR-30c reduced the protein level of NPM1 in EC cells (**Figure 5B**). On the contrary, the inhibition of miR-30c elevated luciferase activity in the wild type 3'UTR group, while it did not affect luciferase activity in the mutant 3'UTR group (**Figure 5C**). In addition, the inhibition of miR-30c increased the protein level of NPM1 in EC cells (**Figure 5D**).

### NNT-AS1 upregulated the expression of NPM1 via sponging miR-30c

**Figure 6:**
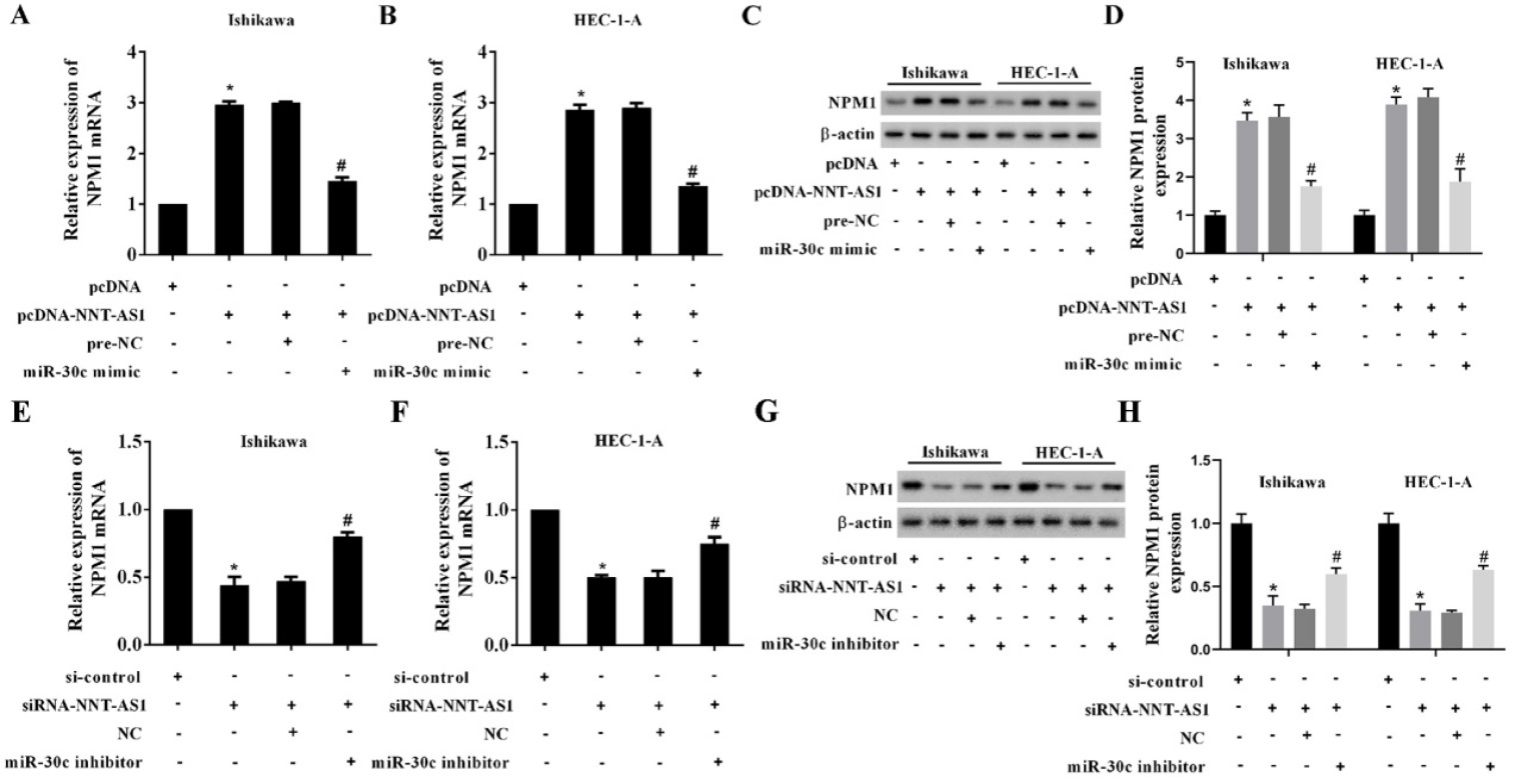
** NNT-AS1 regulated the expression of NPM1 via miR-30c.** Ishikawa and HEC-1-A cells were transfected with NNT-AS1 overexpressing vector (pcDNA-NNT-AS1) or co-transfected with miR-30c mimic. **A.** The mRNA level of NPM1 was detected using qRT-PCR. GAPDH was used as the internal control. **B.** The protein level of NPM1 was detected using Western blotting. β-actin was used as the internal control. Each experiment has three biological duplications. One-way ANOVA followed by Tukey's post hoc test was used for statistical analysis. *p < 0.05, compared with pcDNA. ^#^p < 0.05, compared with pcDNA-NNT-AS1 + pre-NC. Ishikawa and HEC-1-A cells were transfected with NNT-AS1 siRNA (siRNA-NNT-AS1) or co-transfected with miR-30c inhibitor. **C.** The mRNA level of NPM1 was detected using qRT-PCR. GAPDH was used as the internal control. **D.** The protein level of NPM1 was detected using Western blotting. β-actin was used as the internal control. Each experiment has three biological duplications. One-way ANOVA followed by Tukey's post hoc test was used for statistical analysis. *p < 0.05, compared with si-control. ^#^p < 0.05, compared with siRNA-NNT-AS1 + NC.

EC cells were transfected with NNT-AS1 overexpressing vector (pcDNA-NNT-AS1) or co-transfected with miR-30c mimic. According to **Figure 6A-B**, the overexpression of NNT-AS1 increased the mRNA level and the protein level of NPM1, while the miR-30c mimic reversed these effects. In addition, EC cells were transfected with NNT-AS1 siRNA (siRNA-NNT-AS1) or co-transfected with miR-30c inhibitor. The NNT-AS1 interference reduced the mRNA level and the protein level of NPM1, while the miR-30c inhibitor reversed these effects (**Figure 6C-D**). These findings suggested that NNT-AS1 upregulated the expression of NPM1 via sponging miR-30c.

### Interference with NNT-AS1 inhibited the proliferation of estrogen-mediated EC cells via miR-30c/NPM1

**Figure 7:**
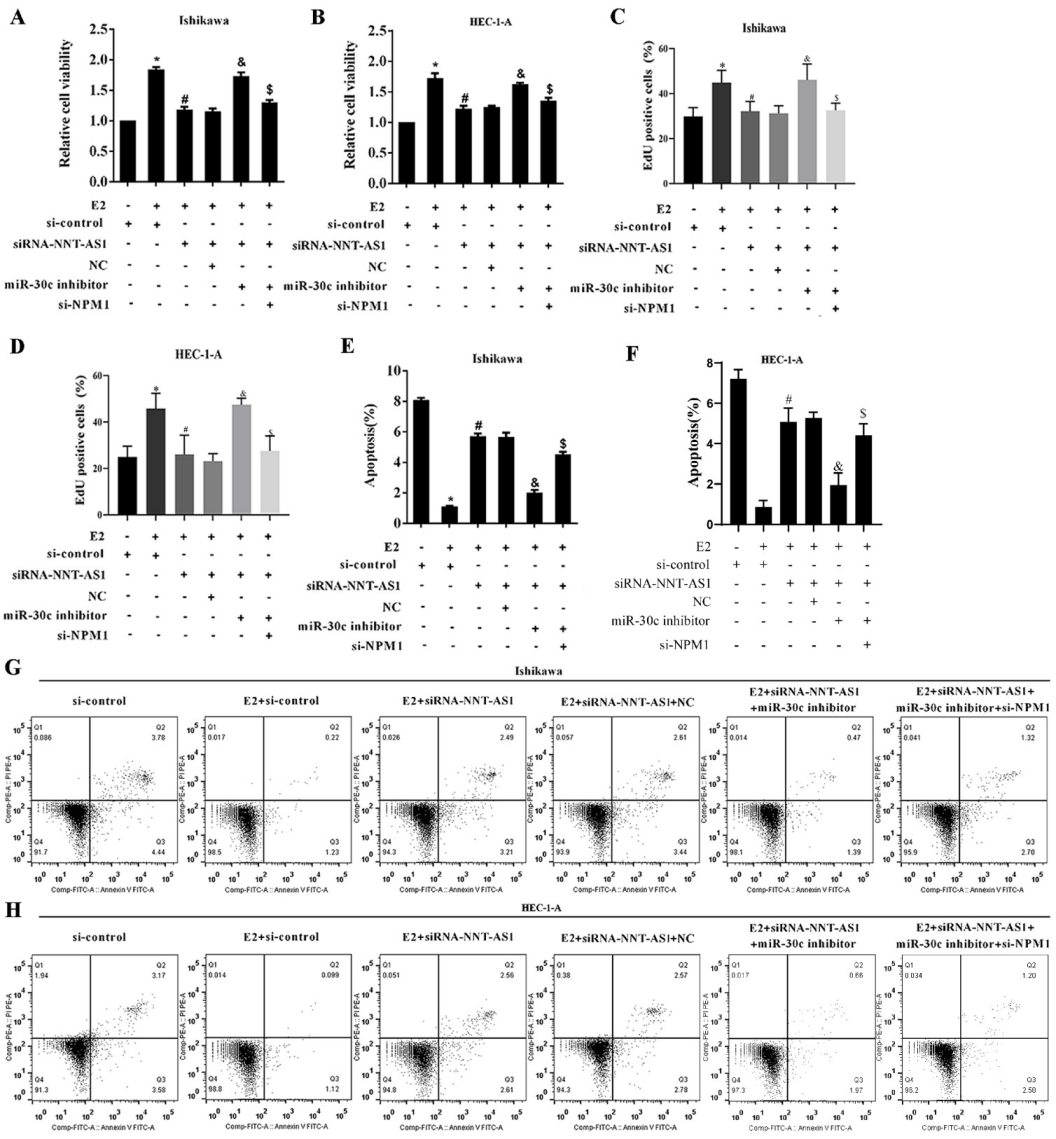
** Interference with NNT-AS1 inhibited the proliferation of estrogen-mediated EC cells via miR-30c/NPM1.** Ishikawa and HEC-1-A cells were transfected with siRNA-NNT-AS1 or co-transfected with NPM1 siRNA (si-NPM1) and/or miR-30c mimic before E_2_ treatment. **A.** The cell viability was detected by Cell Counting Kit-8 assay. **B.** The cell proliferation was detected using the EdU assay. **C-F.** The cell apoptosis was observed by Flow cytometry. Each experiment has three biological duplications. One-way ANOVA followed by Tukey's post hoc test was used for statistical analysis. *p < 0.05, compared with si-control. ^#^p < 0.05, compared with E2 + si-control. ^&^P < 0.05, compared with E2 + siRNA-NNT-AS1 + NC. ^$^P < 0.05, compared with E2 + siRNA-NNT-AS1 + miR-30c inhibitor.

To determine the mechanism of NNT-AS1 on estrogen-mediated EC cell proliferation, we transfected EC cells with siRNA-NNT-AS1 or co-transfected with NPM1 siRNA (si-NPM1) and miR-30c mimic before E_2_ treatment. We found that E_2_ promoted cell viability and proliferation, while siRNA-NNT-AS1 inhibited cell proliferation. Also, miR-30c inhibitor restored cell viability and proliferation, while si-NPM1 reversed these effects (**Figure 7A-B**). Moreover, E_2_ inhibited cell apoptosis, while siRNA-NNT-AS1 increased cell apoptosis. Finally, miR-30c inhibitor reduced cell apoptosis, while si-NPM1 reversed these effects (**Figure 7C-F**). These findings indicated that interference with NNT-AS1 inhibited estrogen-mediated EC cell proliferation via miR-30c/NPM1.

### Interference with NNT-AS1 inhibited the tumor growth of estrogen-mediated EC

**Figure 8:**
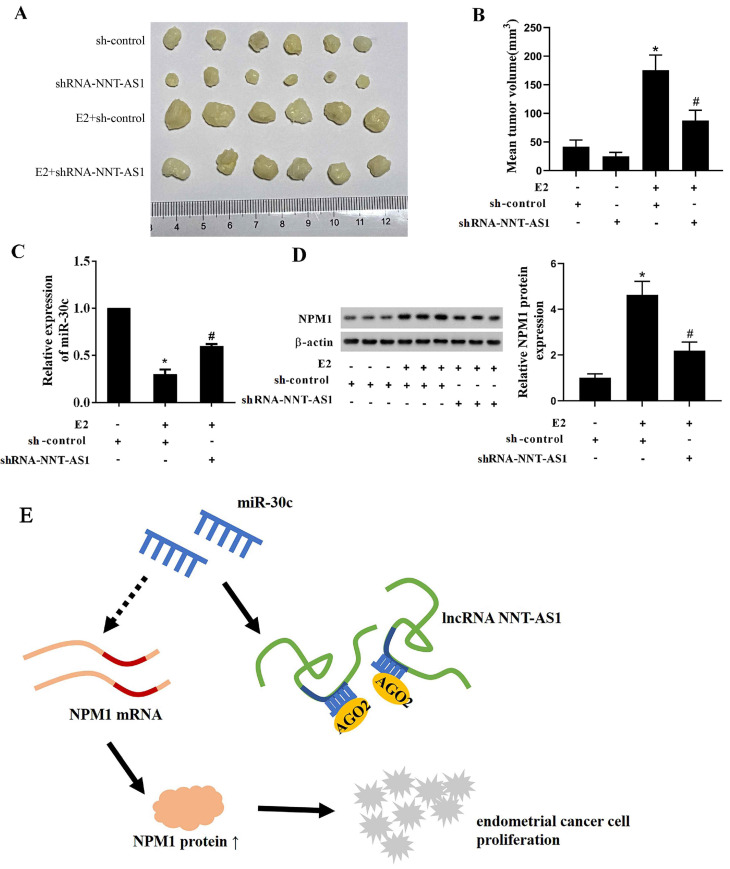
** Interference with NNT-AS1 inhibited the tumor growth of estrogen-mediated EC.** Ishikawa cells stably transfected with sh-NNT-AS1 were injected into nude mice (n = 6 in each group). E_2_ was subcutaneously injected during the tumor growth. A-B. Tumor volume was detected using Vernier caliper. C. The expression of miR-30c was detected using qRT-PCR. U6 was used as the internal control. Each experiment has three technological duplications. D. The protein level of NPM1 in mixture samples of 6 xenograft tissues (n = 3 in each lane) was detected using western blotting. β-actin was used as the internal control. E. Schema depicting the mechanisms of NNT-AS1-mediated EC proliferation. One-way ANOVA followed by Tukey's post hoc test was used for statistical analysis. *p < 0.05, compared with sh-control. ^#^p < 0.05, compared with E2 + sh-control.

To explore whether NNT-AS1 affects tumor growth of E_2_-mediated EC, we injected Ishikawa cells stably transfected with sh-NNT-AS1 into nude mice and subcutaneously injected E_2_ during tumor growth. As shown in **Figure 8A-B**, E_2_ injection significantly increased tumor volume, while interference with NNT-AS1 reduced tumor volume. We also found that E_2_ injection inhibited the expression of miR-30c, while interference with NNT-AS1 increased miR-30c expression (**Figure 8C**). Lastly, E_2_ injection elevated the protein level of NPM1, while interference with NNT-AS1 decreased the protein level of NPM1 (**Figure 8D**).

## Discussion

In the present study, we found that the expressions of NNT-AS1 and NPM1 in EC tissues and EC cell lines were higher than those in benign endometrium tissues and normal EECs. miR-30c expression in EC tissues and EC cell lines was lower than that in delicate endometrium tissues and normal EECs. In EC cells, estrogen increased EC cell viability and proliferation and decreased cell apoptosis, while interference with NNT-AS1 decreased EC cell viability and proliferation and increased cell apoptosis via regulating miR-30c/NPM1 (**Figure 8E**).

To effectively identify and diagnose EC, the underlying molecular mechanisms that contribute to the progression and metastasis of EC have been studied and explored. Numerous studies have shown that lncRNAs are involved in the advancement of EC and become one of the hottest points in cancer studies. For example, Sun *et al.* found that lncRNA HOTAIR could regulate cisplatin-resistant induced autophagy in EC cells 17. Liu *et al.* discovered that lncRNA TUG1 promoted EC development by inhibiting miR-299 and miR-34a-5p 18, and Zhu *et al.* found that lncRNA BANCR promoted proliferation and invasion of EC cells via ERK/MAPK signaling pathway 19. This study focused on the expression and underlying mechanism of NNT-AS1 in EC. We found that the upregulated NNT-AS1 expression was consistent with previous reports 15, 16 that NNT-AS1 acts as an oncogenic gene in colorectal cancer and cervical cancer.

Nowadays, more and more researchers focus on regulatory mechanisms between lncRNAs and miRNAs. LncRNAs might act as miRNA sponges, which could negatively modulate miRNA expression by binding with them and then influence the gene expression that miRNA targeted 20, 21. Our findings revealed that the NNT-AS1 and miR-30c could interact with each other. Such interaction was mediated by AGO2 protein using RIP assay and RNA pull-down assay, consistent with previous regulatory mechanisms between lncRNAs and miRNAs. For example, NNT-AS1 contributed to the cisplatin resistance of cervical cancer by sponging miR-186, thus negating the inhibitory effect of miR-186 on HMGB1 mRNA 22. However, although tumors seemed smaller than the si-control group, it was not statistically significant between the si-control and siRNA-NNT-AS1 groups. We believe that the oncogenic effect of NNT-AS1 might need the support of E_2_, and future studies should investigate these relative mechanisms.

Cancers such as prostate cancer, gastric cancer, squamous cell carcinoma, breast cancer, etc., show aberrant expression of miR-30c 23-25. In 2012, researchers first reported miR-30c in EC 9. They found that EC tissues downregulate miR-30c more than normal endometrial tissues 8, 10. Our study showed that miR-30c was also downregulated in EC tissues, leading to overexpression of NPM1 at mRNA and protein levels. Experimental overexpression of miR-30c decreased both the mRNA and protein levels of NPM1. To further verify the mechanism of NNT-AS1 in regulating estrogen-mediated EC cell proliferation, EC cells were treated with E_2_ and transfected with siRNA-NNT-AS1, miR-30c inhibitor, or si-NPM1. The results suggested that NNT-AS1 promoted EC cell viability and proliferation, and miR-30c reversed this effect. NPM1 also promoted cell proliferation, which proved that NNT-AS1 participated in EC proliferation, thus being involved in the progress of estrogen-mediated EC via miR-30c/NPM1. We found nine possible NNT-AS1 targets in the preliminary experiment, including miR-320a, miR-203, miR-22, miR-424, miR-363, miR-142-3p, miR-129-5p, and miR-186, through biological information prediction analysis and literature review. The RNA pull-down experiment found that HEC-1-A cells, miR-30c, miR-320a, miR-22, and miR-424 were enriched in the NNT-AS1 pulled-down complex (**Supplemental Fig. 1**). As the fold enrichment of miR-30c is significant, miR-30c was chosen as the candidate in the current study. The effects of the other three candidate miRNAs, including miR-320a, miR-22, and miR-424, are under investigation by our research group. The results are not yet available.

## Main findings and Conclusions

In conclusion, these results suggest that NNT-AS1 affected the proliferation of estrogen-mediated EC by regulating miR-30c/NPM1. This research first revealed the role of NNT-AS1 in the expansion of EC and confirmed a potential mechanism that NNT-AS1 mediated, which may provide molecular targets for the treatment of EC.

## Supplementary Material

Supplementary figure.

## Figures and Tables

**Table 1:** Correlation of NNT-AS1 expression with different clinicopathological features of endometrial cancer

Clinicopathologic parameters	N	NNT-AS1 expression		*p* value
		Low (%)	High (%)	
**Age (years)**				
≤ 55	10	6 (60.0%)	4 (40.0%)	0.465
>55	30	14 (46.7%)	16 (53.3%)	
**Pathology type**				
endometrioid adenocarcinoma	29	13 (44.8%)	16 (55.2%)	0.288
other pathology types	11	7 (63.6%)	4 (36.4%)	
**FIGO stage**				
I-II	26	17 (65.4%)	9 (34.6%)	0.010
III- IV	14	3 (21.4%)	11 (78.6%)	
**Pathology classification**				
G1	22	16 (72.7%)	6 (27.3%)	0.006
G2	11	3 (27.3%)	8 (72.7%)	
G3	7	1 (14.3%)	6 (85.7%)	
