# Supplementary material for: Long non-coding RNA NNT-AS1 positively regulates NPM1 expression to affect the proliferation of estrogen-mediated endometrial carcinoma by interacting
Source: J Cancer. 2022 Jan 1;13(1):112–23. doi: 10.7150/jca.62630 (PMC8692688; doi:10.7150/jca.62630)
Supplement: Supplementary file 1 — Supplementary figure. [file jcav13p0112s1.pdf]

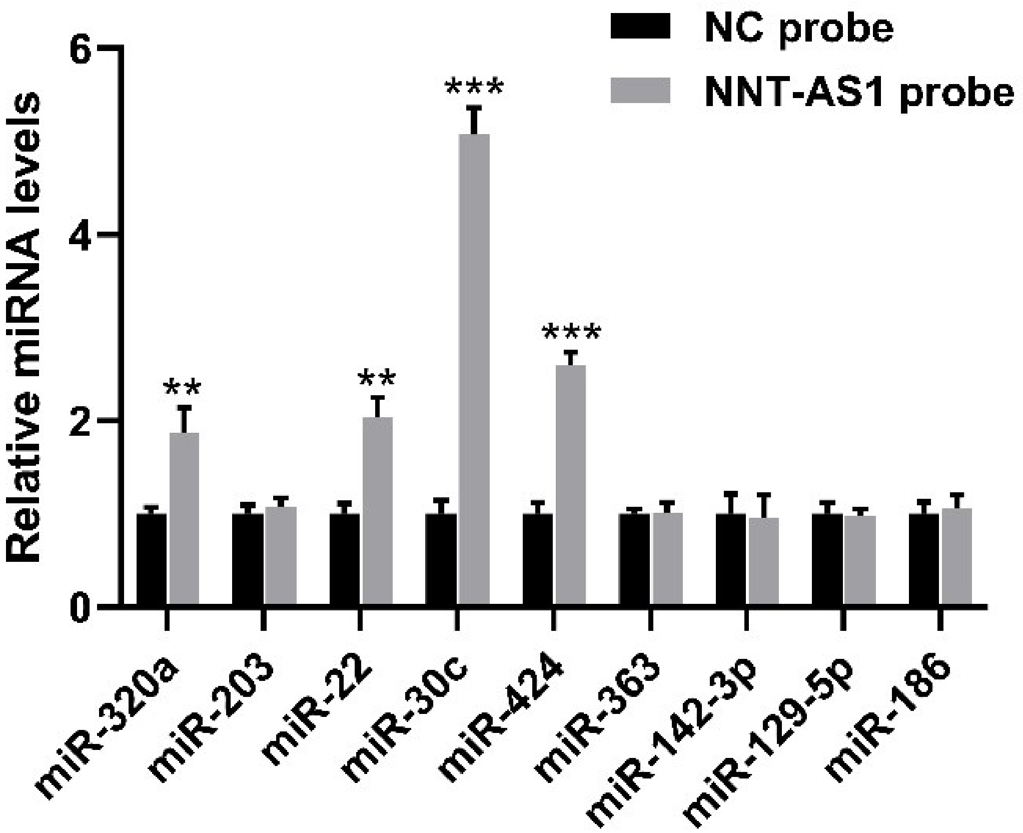

Supplemental Figure 1. Candidate miRNAs that may interact with NNT-AS1. The RNA pull-down experiment found that in HEC-1-A cells, miR-30c, miR-320a, miR-22, and miR-424 were enriched in NNT-AS1 pulled-down complex. One-way ANOVA followed by Tukey's post hoc test was used for statistical analysis. $**p < 0.01$, $***p < 0.001$, compared with NC probe.
